# Cingulotomy: the last man standing in the battle against medically refractory poststroke pain

**DOI:** 10.1097/PR9.0000000000001149

**Published:** 2024-03-22

**Authors:** Linda Kollenburg, Erkan Kurt, Hisse Arnts, Saman Vinke

**Affiliations:** a Department of Neurosurgery, Radboud University Medical Center, Nijmegen, The Netherlands; b Donders Center for Brain, Cognition and Behaviour, Department of Neurosurgery

**Keywords:** CPSP, Cingulotomy, Stroke, Central pain, ACC

## Abstract

**Introduction:**

Central poststroke pain (CPSP) places a huge burden on patient lives because patients are often refractory to conventional strategies and have little chance for spontaneous recovery. A subset of patients is even given approval for euthanasia and is without any perspective. Because the anterior cingulate cortex historically seems to be a promising target for patients with both mental and chronic pain disorders, lesioning of this central “hub” with cingulotomy may be a useful strategy for medically refractory CPSP. However, limited research is available on cingulotomy for central pain. Hence, we represent a rare case in which cingulotomy is performed on a patient with CPSP.

**Objectives:**

To describe the potential of cingulotomy in a case with CPSP.

**Methods:**

The case presented in this study concerns a 60-year-old woman who experienced CPSP, caused by a hemorrhagic stroke in the basal ganglia and thalamus. The patient visited several centers and tried multiple off-label treatments; however, she was told nothing else could be done and was even given approval for euthanasia. Hence, anterior cingulotomy was performed.

**Results:**

After surgery, no transient adverse events occurred, except for vocabulary disturbances post stroke, which disappeared after several weeks. After 14 weeks, changes in pain behavior were observed, followed by a decreased pain intensity. At a later follow-up, the pain had completely disappeared.

**Conclusion:**

Anterior cingulotomy seems to be a suitable “last-resort” option for patients with CPSP. Future research, including homogenous groups, to define the best location for lesioning is required to allow the revival of this “old” technique in the current era.

## Abstract 2

Cingulotomy might be a suitable “last-resort” treatment option for medically refractory central poststroke pain due to its success on mental and chronic pain disorders.

## 1. Introduction

Every year, on average, 16.9 million people worldwide experience a stroke, with up to 35% experiencing a form of central poststroke pain (CPSP).^3,5^ Central poststroke pain can manifest as contralateral localized pain in the extremities, face, abdomen, and thorax or in the hemibody with descriptions varying from aching, dull, and throbbing to sharp, stabbing, shooting, and/or burning forms of pain.^28^ Central poststroke pain has been described as “among the most distressing, incapacitating, and intractable pain syndromes.”^16^ Despite the presence of a wide variety in treatments for chronic pain, most patients with CPSP remain refractory to these strategies and thus resort to drugs, such as high doses of morphine, or off-label cannabis and marijuana in a desperate attempt for pain relief.^16^ Nevertheless, a subset of patients remains without perspective and is even approved for euthanasia. Because these patients have little chance for spontaneous recovery, alternative neurosurgical approaches (eg, cingulotomy) should be considered. Historically, cingulotomy has also been performed for a variety of mental diseases and later also for chronic pain syndromes.^13,31^ In anterior cingulotomy (ACING), lesions are created in the anterior cingulate cortex (ACC), which is known to be involved in cognitive and emotional processing of pain signals.^24^ The potential for destructive lesioning of this central “hub” in pain processing was confirmed when ACING caused pain relief in 60% of patients with chronic pain, with pain being described as “not particularly bothersome” after surgery.^13,26^ However, its use in practice, especially for patients with central pain, has been rather limited. To our knowledge, only 4 cases with CPSP, undergoing cingulotomy, have been described.^19,23^ The destructive nature and uncertain mechanism of this technique likely contributed to this phenomenon. However, because historical evidence shows that the ACC seems to be a suitable target, we present a rare case of this relative “forgotten” technique that belongs to the standard neurosurgical arsenal and show its efficacy in a patient with refractory CPSP.

## 2. Methods

**Figure 1.**
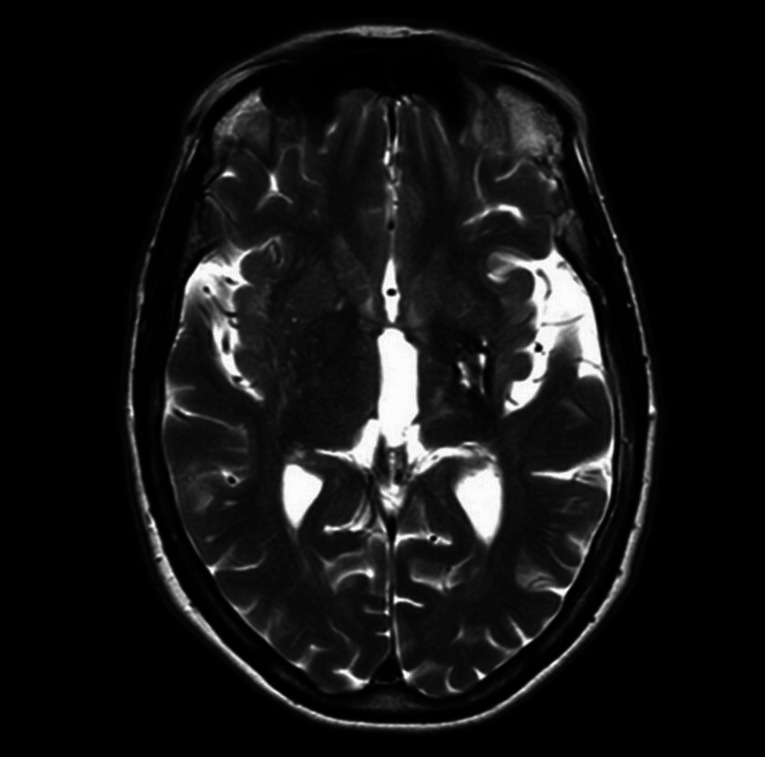
Axial magnetic resonance image showing poststroke changes in the left and right hemisphere.

Illustrative case: In 2014, a 60-year-old woman experienced a hemorrhagic stroke in the basal ganglia and thalamus on the left side (Fig. 1). Post stroke, the patient experienced hemiparesis on the right side and, after 4 months, she developed right-sided hemibody pain. The pain was present continuously in the face, arm, thorax, abdomen, and leg. Sensations ranged from sharp, bursting, burning, cold, and exhausting forms of pain with an average NRS score of 9 and some pain peaks reaching the maximal NRS score. During activities of daily living and pressure-increasing moments, the pain intensity increased, whereas warmth, massage, walking, exercise, and relaxation reduced the pain. Since 2014, the patient received various pain medications including amitriptyline, morphine, zaldiar, pregabaline, and tapentadol, as well as various off-label treatments, including a trial block of the stellatum ganglion, botox injections, and a ketamine infuse. Even antispasmodic medications such as baclofen and tizanidine were prescribed. Despite undergoing all these treatments, the pain became unmanageable. Physicians told her nothing else could be done, and she was even given approval for euthanasia in 2019. As a last resort, the patient presented the same year at our department due to our expertise in the field of neuromodulation and stereotaxy. We have considered motor cortex stimulation (MCS); however, because pain was dispersed in the hemibody and not specifically limited to the face and/or arm, and the leads available for MCS being too small to cover all the different motor cortex areas involved, MCS was hypothesized to cause insufficient pain relief. Deep brain stimulation of the ACC (DBS-ACC) has also been considered. However, it was expected to only be beneficial in the long-term because settings (eg, voltages) are adjustable and it often takes time for patients to find the best setup. When considering the urgency of immediate pain relief for this patient and her approval for euthanasia, DBS-ACC was not selected. After several multidisciplinary discussions with local members of the pain team, ethics officers, primary care physician, psychologist, and physiotherapist, ACING was selected due to the presence of an affective emotional component and its previous success rates for various other chronic pain conditions. The patient gave written informed consent to the procedure and usage of her data for this report.

## 3. Surgical technique

**Figure 2.**
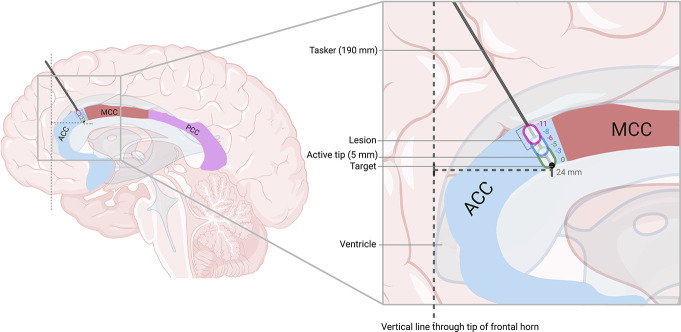
Anterior cingulotomy with lesioning from the target in the ACC with withdraw of 2/3 mm after each lesion, using a tasker lesion electrode with a length of 16 mm and active tip of 5 mm. ACC, anterior cingulate cortex; MCC, medial cingulate cortex; PCC, posterior cingulate cortex.

**Figure 3.**
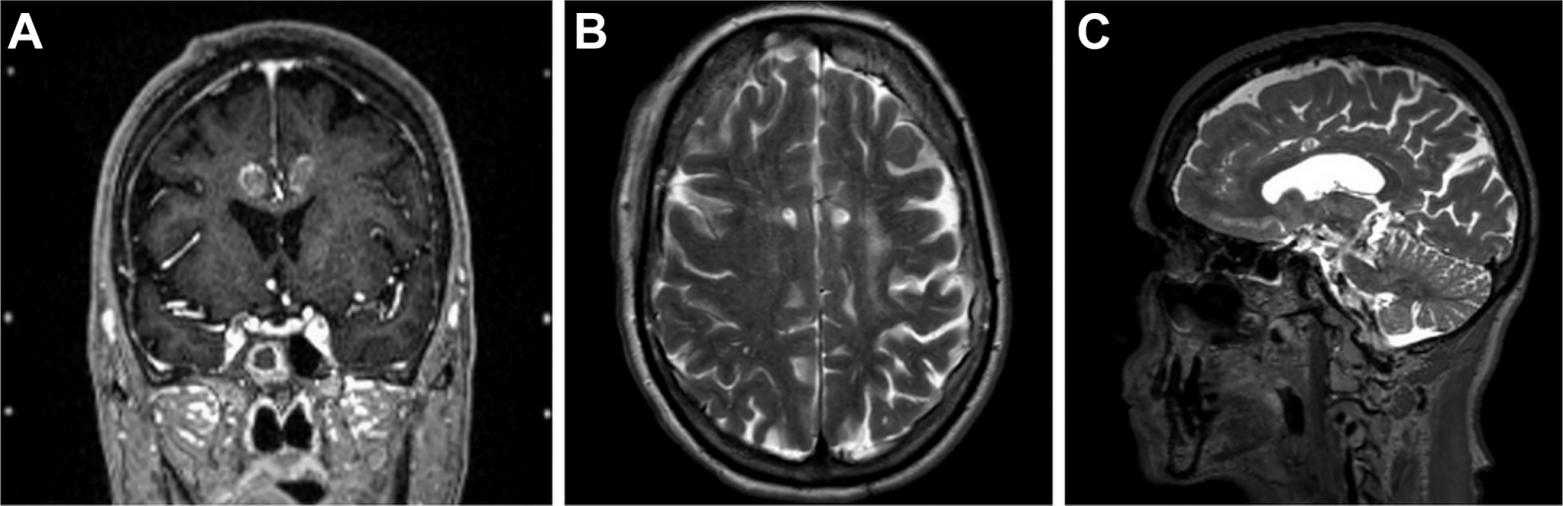
Magnetic resonance images of bilateral anterior cingulotomy in the coronal plane (A) intraoperative, and in the axial (B) and sagittal plane (C) one-year postoperative.

The surgery was performed under general anesthesia using a stereotactic frame. Surgical planning was performed with the stereotactic planning software of Brainlab. Anterior cingulotomy was performed in the dorsal region of the ACC (dACC). Targeting of the cingulate tract was based mainly on visual anatomical knowledge, respecting the vascular anatomy of the patient. The target corresponded with 24 mm posterior from the frontal horn and 6 mm laterally from the midline (Fig. 2). In total, 2 burr holes were drilled into the skull with an 8-mm disposable trepan. Afterwards, lesioning was performed using a tasker lesion electrode with a length of 190 mm and active tip of 5 mm (Diros Technology inc, Ontario, Canada). After each lesion, the tasker was withdrawn 2/3 mm, thereby allowing optimal cross-sectional coverage of the dACC from the target (Fig. 2). With this technique, 2 lesions (on target and −3 mm) and 3 lesions (on target, −3 mm, and −6 mm) were created on the left and right sides, respectively (Figs. 2 and 3). The difference in the amount of lesions created on each side is partially caused by the presence of a large blood vessel near the dACC, making it unsafe to perform a third lesion at the left side. Furthermore, the asymmetrical anatomy of the cingulum also prevented additional lesioning at the left side because the right side was larger in diameter. For each lesion on both sides, the tip was heated to 75 °C for 60 seconds with a ramp time of 15 seconds. The tasker was not removed from the site of the lesion until the temperature was decreased to 37 to 38°.

## 4. Results

**Figure 4.**
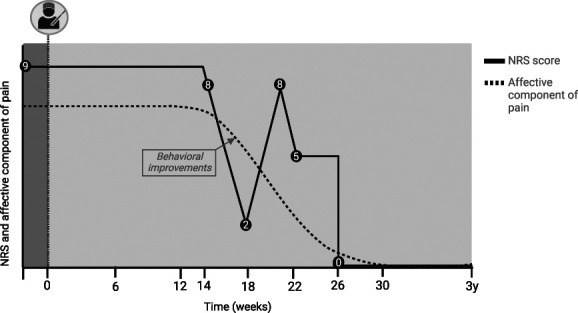
Perioperative change in NRS score and affective component of pain. The dark and light gray fields indicate the preoperative and postoperative period respectively. The shape of the dashed line does not represent the actual change in affective component of pain, but was drawn to illustrate the onset of changes at 14 weeks.

Following ACING, common adverse events (AEs) including transient urinary incontinence, transient confusion, and changes in affect remained absent. Before surgery, the patient already showed vocabulary disturbances post stroke; these, however, progressed post operation because she experienced even more difficulty when trying to find the correct words while speaking. The patient did not have similar problems when texting and writing. Immediately and several weeks post operation, the NRS score remained unchanged (Fig. 4). After 14 weeks, major behavioral improvements including decreased crying and sadness, and improved interaction with surroundings, were present. In the following 8 weeks, the pain varied between NRS 2 and 8 (Fig. 4). Though moments of high pain scores were present, these would rapidly cease to lower intensities. Despite the patient realizing the presence of the pain, even with high NRS scores at times, she was no longer bothered by it and even said to feel “a completely new person.” Gradually, the pain decreased with a complete disappearance of pain and recovery of vocabulary after 26 weeks (Fig. 4). After 3 years of follow-up, the pain remained absent, and she even said to regret ever asking for euthanasia: “My life has changed significantly and I look bright into the future now”.

## 5. Discussion

This rare case illustrates that ACING is a promising “last-resort” therapy for those experiencing CPSP, even when euthanasia has already been approved and all other options have failed. Whereas the treatment of central pain often focuses on pain intensity alone, ACING also targets factors involved in suffering, including emotional reaction, attention, processing, and perception of pain. The ACC seems to be an important limbic structure in various chronic pain conditions because it is most frequently connected to pain processing in all cortical areas and regulates the affective content and motor response selection of noxious stimuli.^4,20^ In patients with various chronic pain conditions, including CPSP, enhanced excitatory synaptic neurotransmission and Aβ-fiber–mediated activation, dendritic dysfunction, decreased inhibition and opioid receptor binding, increased intrinsic cellular excitability, and altered somatosensory processing were seen in the ACC, hence supporting its potential as a neurosurgical target for central pain.^18,21,22,25,33,36^

The efficacy of ACING was found to be 50%-100% at different follow-up moments for patients with various pain syndromes of benign origin.^7,9,10,13,17,23,29,30,32,34^ Of interest, only a total of 4 patients with CPSP were included in these studies, hence contributing to the rareness of the case presented in this study. One of these patients with CPSP was included by Pillay and Hassenbusch, in which significant pain relief was reached in 60% of patients with chronic intractable pain.^23^ However, their study population was heterogeneous; hence, conclusions should be taken with care. The study by Kim et al. included a homogenous group consisting of 3 patients with CPSP and observed a 51.9% decrease in the VAS score.^19^ However, the subjects by Kim et al. had also received ventralis caudalis deep brain stimulation (DBS), which possibly interfered with the results observed for cingulotomy.^19^ Similar to our case, major improvements in quality of life were seen due to the effects on pain behaviour and intensity. These results were also expected considering the role of the ACC in pain perception and/or processing and the previous success rates of ACING for other chronic pain disorders. The effects observed in the current and previous cases of central pain are hypothesized to be caused by restoration of the balance of the limbic system because it possibly compensates for the hyperactivated ACC.^14^ Similar to our case, a delayed effect of ACING has also been observed in patients with obsessive-compulsive disorder, hence arguing against any placebo effect.^2,35^ It is hypothesized that secondary neural degeneration and/or metabolic alterations in brain areas surrounding the lesions might be responsible for this phenomenon.^8^ By contrast, other studies reported faster responses following ACING for patients with cancer pain and CPSP.^19,27^ We have performed ACING with great care using our best expertise and most advanced technologies. Nevertheless, the occurrence of a single AE, which included difficulties in vocabulary, could not be prevented. On reviewing of the previous reports, aphasia and/or inappropriate language seemed to be present in 2% (4/236) of patients with chronic intractable pain undergoing cingulotomy.^26^ Despite the absence of an explanation for this AE, the presence of aphasia in our case pre surgery is likely to have contributed to this occurrence. Of interest, in all reports, cases recovered in speech after a matter of hours to several weeks.

The introduction of less-invasive pharmacological treatments for pain as well as advanced neuromodulation techniques, probably contributed to the neglection of this “old” technique in the current era.^26^ Variability in the effectivity of ACING is likely caused by the inclusion of heterogenous groups. Besides, usage of various definitions for “responders” complicated proper analysis of previous study outcomes. While some authors use cognitive alterations in pain behavior to define “responders,” others use reduction in the VAS score or categories (eg, significant, good, and excellent) instead. Despite the absence of a standardized surgical protocol for ACING, we have performed lesioning in the dorsal portion of the ACC because most other reports mention a similar target. Before the selection of cingulotomy as treatment for our patient, we also considered DBS. Though evidence on DBS for chronic pain remains limited, various targets for stimulation have been tried including, the cingulum (DBS-ACC), thalamus, and periaqueductal gray.^15^ Regarding DBS-ACC, its results have not specifically been defined for CPSP; however, it was found to cause substantial pain relief in 50% of patients experiencing other chronic pain disorders.^11^ Despite both cingulotomy and DBS-ACC having a similar target and indication, various aspects should be considered when comparing these approaches. Whereas lesioning causes irreversible destruction of neurons, DBS-ACC allows for reversible and personalized modulation. Despite irreversible destruction causing cingulotomy to have a higher chance on permanent side effects, it is suggested to be more effective because the DBS electrode (1.27 mm) is much smaller than the lesions (1.2–2 cm) created with cingulotomy.^1,6,12^ Furthermore, unlike for cingulotomy, DBS-ACC is more costly because it requires implantation of expensive devices and reimbursement is not guaranteed in most countries. Taking all these factors into consideration, ACING is still a well-suited, probably highly effective and low-cost operative treatment strategy for patients with CPSP who are left without any perspectives needing urgent pain relief.

## 6. Conclusions

Based on the current results, it can be concluded that ACING is a suitable “last-resort” option for patients with CPSP who have reached the end of the spectrum of traditional pain treatments. Because not only physical sensation but also psychological and emotional components are involved in the pathophysiology of CPSP, more research should be dedicated to this “old” technique, to allow its revival in the current era. However, future research using homogenous groups is necessary to define the best location for lesioning.

## Disclosures

The authors have no conflict of interest to declare.
